# Sex-specific but not urbanisation-related behavioural differences in a wolf spider, *Pardosa alacris*

**DOI:** 10.1038/s41598-026-41239-2

**Published:** 2026-03-05

**Authors:** Tibor Magura, Roland Horváth, Szabolcs Mizser, Mária Tóth, Ferenc Sándor Kozma, Gábor L. Lövei

**Affiliations:** 1 Department of Ecology, Faculty of Science and Technology, University of Debrecen, Egyetem sq. 1, Debrecen, H-4032 Hungary; 2 HUN-REN–UD Anthropocene Ecology Research Group, Egyetem sq. 1, Debrecen, H- 4032 Hungary; 3 Count István Tisza Foundation for the University of Debrecen, Egyetem Sq. 1, Debrecen, H-4032 Hungary; 4 Juhász-Nagy Pál Doctoral School of Biology and Environmental Sciences, University of Debrecen, Egyetem sq. 1, Debrecen, H-4032 Hungary; 5 Department of Agroecology, Flakkebjerg Research Centre, Aarhus University, Slagelse, DK-4200 Denmark

**Keywords:** Exploratory, Locomotory activity, Personality, Risk-taking, Rural, Urban, Urban ecology, Behavioural ecology

## Abstract

Urbanisation, one of the main driving forces of the decline in arthropod diversity, is a global environmental problem. Urbanisation causes changes to the size, connectivity, structure, and environmental parameters of their natural habitat. Due to a host of novel conditions and situations, high exploratory and risk-taking behaviours are beneficial traits to cope with urban environments. Therefore, we hypothesised that urban spiders should display more exploratory and risk-taking behaviour than their rural conspecifics. We tested 253 individuals of a widespread, forest-associated ground-dwelling wolf spider species, *Pardosa alacris*, sampled from rural and urban forest sites during their peak activity period, for their locomotory activity, exploratory and risk-taking behaviour by six frequently used behavioural measures. Combining the studied behavioural measures into composite scores using redundancy analysis, we identified two composite variables, the activity-exploration-boldness and the risk-taking behavioural ones. Behaviour measured by the composite activity-exploration-boldness score was significantly repeatable, but not the composite risk-taking behavioural one. There were no urbanisation-related differences in the composite behavioural scores, suggesting that higher exploratory or risk-taking behaviour may not yield fitness benefits in this generalist predator. We found, however, significant sex-specific differences in the composite activity-exploration-boldness behavioural scores. The higher activity, exploratory and boldness in males than females may be explained by their different life-history strategies and sex-specific selective pressures.

**Supplementary Information:**

The online version contains supplementary material available at 10.1038/s41598-026-41239-2.

## Introduction

Urbanisation is one of the most recent and fastest anthropogenic processes transforming the Earth’s biota^1,2^. While urbanisation progresses through various steps and displays regional differences in extent, intensity, energy use and resource concentration, it has the overall common outcome of replacing a natural environment with one that creates several important new environmental conditions^1^. Animals trying to survive in such environments have to cope with novel challenges, ranging from increasing frequency of human disturbance to exposure to pollutants^2^. Responses can be various, but behavioural ones are often the first to altered conditions^3^. Boldness, high exploratory activity and aggressiveness can especially be useful for coping with novel challenges in urban habitats^3^.

Personality traits in arthropods, similarly to other animals - defined as consistent behavioural differences among individuals - can have varied effects on their fitness^4^. Bold individuals may be more effective at exploring new environments or finding food, while shy individuals might excel at avoiding predators^5^. In male field crickets (*Gryllus campestris*), bolder individuals are more likely to secure mating opportunities, but at the cost of higher risk of predation^6^. Aggressiveness can also increase mating success, while more cautious individuals may have higher survival rates and avoid risky behaviours that could lead to death before reproduction. This trade-off between reproductive success and survival demonstrates how personality traits can influence fitness, and thereby affect population persistence and adaptive responses to environmental challenges^7^. Bolder individuals of the orb spider (*Metellina mengei*) are more likely to build webs in exposed areas with high prey availability but face higher predation risk^8^. Urban individuals of the orb-weaving spider (*Larinioides sclopetarius*) are bolder and more exploratory than rural ones^9^. In cities, these spiders often build webs on buildings and bridges, exposed to artificial light, and thus benefit from insect prey attracted to lights.

Animals living in urban environments may experience reduced predation pressure due to a lack of natural predators^10^. Indeed, in urban habitats, the abundance of natural enemies of ground-dwelling arthropods (e.g. birds, reptiles, and mammals) may be reduced^11^. This can lead to a decrease in anti-predator behaviour, as there is less need for vigilance. Thus, in urban settings, where predation risk is lower, arthropods may prioritise foraging and mating over predator avoidance, which can alter their overall personality profile^12^.

Urban environments are often subject to several forms of disturbance, yet some arthropods have evolved to become less sensitive to such stressors^3^. Reduced behavioural responses to stress can lead to more consistent behaviours in urban settings, allowing arthropods to maintain normal functioning despite environmental disruptions. Indeed, urban orb-weaving spiders remain in their webs even when disturbed^13^.

There are several behavioural traits that make arthropods successful in urban habitats^14^. Bold, exploratory individuals are more likely to disperse into urban areas and establish populations there. Over time, this can lead to an increase in the frequency of these behavioural traits in urban arthropod populations.

Sex is also an important factor when assessing behavioural traits, because sex-specific differences in boldness and exploration are also widespread across animal taxa and are often linked to divergent reproductive strategies and life-history trade-offs^15^. In many species, males tend to exhibit higher levels of risk-taking and exploratory behaviour, which is commonly interpreted as an adaptive response to sexual selection pressures favouring increased mate-searching activity^15^. In contrast, females are frequently more risk-averse, potentially reflecting stronger selection for survival and securing future reproductive output^15,16^.

In order to examine whether urbanisation selects for specific behavioural traits in a common spider, we examined whether (a) behavioural consistency exists in a common wolf spider (*Pardosa alacris*), (b) whether rural- vs. urban-living adults of this species show behavioural differences, and (c) whether there are sex-specific differences between adult males and females.

## Materials and methods

### Study area

The study area was in and around the city of Debrecen (47°32′ N; 21°38′ E), the second largest city of Hungary (199,858 inhabitants in 2022, and an area of 461.7 km²). The city is bordering the biggest (1092 ha) mature (> 120 years) lowland oak forest (*Convallario-Quercetum roboris*) on the Great Hungarian Plain, which has been protected since 1992 and is part of the Natura 2000 network (site code: HUHN20033, with a total area of approximately 1000 ha) since 2004. From the early 1900 s, the southern part of the forest has been fragmented by the expanding city, creating several isolated fragments. This provides an excellent opportunity to study urbanisation-driven patterns and processes in forest sites with similar composition and history, as well as meso-climate, topography, and soil parameters.

To study the ecological effects of urbanisation, four mature rural and four urban sites, dominated by English oak (*Quercus robur*) were selected (see https://data.mendeley.com/datasets/ptmzyr74zx/1 for a KML file showing the location of the sites). The size of these sites was similar (mean area of the rural and urban sites was 3.61 ha and 3.60 ha, respectively), and large enough to maintain self-supporting, habitat-specific arthropod assemblages, that did not depend on immigration from the surrounding matrix. The mean distance between sites was 396.5 m and 702.2 m for rural and urban sites, respectively, with a minimum distance of 2,559 m between a rural and an urban site. The proportion of built-up and impervious surfaces was significantly higher around the urban sites than rural ones (60 ± 5.4% vs. 0 ± 0%, measured within 1000 m radius). Furthermore, the intensity of both the management interventions (no regular interventions in the rural sites vs. intensive shrub thinning in urban ones) and trampling (minimal in rural vs. substantial in urban) was considerably different. Significant urbanisation-associated changes in several environmental parameters were confirmed, including higher soil and soil surface temperatures, higher soil pH, higher soil calcium and zinc concentration, and less coarse woody debris at urban than rural sites^17^.

### Model organism and sampling design

For this study, a ground-dwelling wolf spider, *Pardosa alacris* C.L. Koch, 1833 (Araneae: Lycosidae) was selected. *P. alacris* is a widespread western Palaearctic species, present in almost all of Europe, across the Caucasus (Russia), to as far east as Kazakhstan. In Central Europe this ground-dwelling active hunter^18^ is very common in deciduous forests with dense leaf litter^19^, but also occurs in exotic pine plantations^20^. Immature *P. alacris* emerge after overwintering during late March-early April, when they start their adult moult. Males are active until the end of June, and females until the end of September, when the peak of their activity occurs. The peak activity of juveniles coincides with that of the females. Juvenile spiders are active until November, when they start overwintering^21^. In the studied mature lowland oak forest, *P. alacris* is a dominant ground-dwelling species, showing no significantly decreased abundance in urban sites^22,23^.

Spiders were collected live using unbaited pitfall traps during the main activity periods of females and males, from late March to the end of June, 2020. A total of 120 pitfall traps were installed (2 areas × 4 sites × 15 traps). Traps were identical in material and arrangement as described in Magura et al.^24^. We collected a total of 253 adult *P. alacris*; 63 females and 85 males at rural sites, while 47 females and 58 males at the urban ones (Table S1). Trapped spiders were transported to the laboratory, sexed, and their live body mass was measured (two repeats, precision 0.1 mg). After weighing, spiders were individually kept in a Petri dish (90 mm diameter) lined with wet filter paper until the start of the behavioural tests. Before the tests, only water was provided because both satiation and dehydration could influence their behaviour.

*P. alacris* is not a protected species and it is not under ethical regulation in Europe (Directive 2010/63) or in Hungary. The experiments were nevertheless carried out in accordance with the animal experimentation guidelines of the Ethics Committee of the University of Debrecen. For the study, only the minimal number of spiders necessary to obtain reliable and robust results was used. During the behavioural tests, the spiders were handled carefully to minimise stress. After the experiments, all spiders were released back into the study areas.

### Behavioural tests

In the laboratory, spiders were kept under standardised conditions (24 °C, 40% relative humidity, natural L: D cycle). After transporting from the field, spiders had a 2 h of acclimatisation and resting period before beginning the behavioural tests. First, locomotory activity, boldness and exploratory behaviour were measured in a novel environment test, also called an open field test^14,24,25^. Immediately afterwards, we tested the responses to threats, and escape behaviour in an arena^24,26,27^. Experimenters were not aware of the origin of the individual tested. Both tests were repeated after a 24 h resting period.

The reaction to a novel environment was studied using an open white plastic box (364 × 230 mm), the bottom of which was divided into 35 equally sized squares^27^. At the beginning, a randomly selected spider was placed in the central square and covered with a Petri dish (diameter 55 mm). When the spider stopped moving (did not move for 5 s), the lid was swiftly lifted, taking care not to touch the individual, and its movements were recorded for 90 sec^14^ using a GoPro HERO6 camera (CHDHX-601-FW). The video recordings were analysed using the Windows Movie Maker video editor software (version 8.0.7.5.) and the following measures were recorded: (1) the number of squares covered by the individual (henceforth referred to as *no. squares visited*), which is a measure of locomotory^26,28^ or exploratory behaviour^14^; (2) the number of squares entered that were not adjacent to the arena wall (*no. inner squares visited*), a measure of exploration^26^ or boldness^28^; (3) the time it took the spider to reach the wall (*time to wall*), a measure of exploratory behaviour^29^ or boldness^28^; and (4) the time the individual spent in the squares adjacent to the wall (*edge preference* hereafter). This is also called “centrophobism”, and is an inverse measure of exploratory behaviour or boldness^26,30^.

The escape behaviour of spiders in response to a simulated attack was tested in a ring-shaped arena divided into eight equal segments^26^ (Fig. S1). Spiders were individually placed in the arena and allowed to habituate. When they stopped moving, the spider was gently hit on its back with a small forceps. The test ended once the individual stopped moving^26^. We recorded: (5) the time spent running (henceforth *escape duration*) and (6) the number of segments crossed during the escape (*escape distance*).

Arenas were cleaned with 70% ethanol after every fifth test but sooner if the tested individual defecated or left any clues (e.g. silk). We have no clear evidence that conspecifics leave behind chemical cues that influence spider behaviour but if this exists, the fact that we did not clean the arena after every single test could potentially confound the results. Repeatability was tested by conducting all behavioural tests twice, with 24 h between them^26,30^. Testing only twice prevented any habituation to the experimental conditions, and there is no evidence that repeatability increases with the number of trials^31^.

### Statistical analyses

All statistical analyses were completed using the R program environment^32^. The behavioural measures evaluated were highly correlated (Table S2), effectively reducing the six behavioural measures to two axes. Therefore, the six behavioural measures were combined into composite variables using redundancy analysis (RDA)^33^ using the *vegan* package^34^. Prior to ordination, all behavioural variables were standardised to zero mean and unit variance to account for differences in measurement scales. Correlation between the behavioural measures and the RDA axes was tested with the *envfit* function in the *vegan* package (with 999 permutations). Convex hulls and their areas around the points of the rural and urban individuals on the ordination plot were also calculated and visualised using the *vegan* package.

Since the quantity of the food consumed, and thus the body mass, can influence the behaviour of arthropods^28^, we first tested for any significant effect of body mass on the composite behavioural scores derived from RDA with linear mixed models (LMMs), setting the habitat type (rural vs. urban sites), sex (female vs. male), the body mass and their interactions as explanatory variables using the *lme4* package^35^. Given that body mass had no significant effect on the composite behavioural scores, either separately or in interaction with habitat type and sex (Table S3), body mass was not incorporated in the subsequent LMMs. The effects of habitat type, sex, and their interaction on the composite behavioural scores were also analysed with LMMs. Before the LMMs, the best fitted probability distribution of our response variables was identified with the help of the *car*^36^ and *MASS*^37^ packages. Based on these tests, the composite behavioural scores were modelled using a normal error distribution^38^. The nested design (sites nested within habitat) was also taken into account and included as a random factor. The trials were regarded as repeated measures, while the experimenter, as well as the spider identity (ID) were added as random factors. Conditional and marginal R^2^ values for the LMMs were checked by the *MuMIn* package^39^. Model assumptions were checked by inspecting residual normality with the Q-Q plots using the *car* package.

To test whether individual spiders tended to rank similarly across composite behavioural scores, the Kendall’s coefficient of concordance was calculated using the *DescTools* package^40^. To test consistency between trials, repeatability from the LMMs with the individual IDs as a random term was estimated using the *rptR* package^41^ first for all individuals, then for females and males, and finally separately for rural and urban spiders.

## Results

**Fig. 1:**
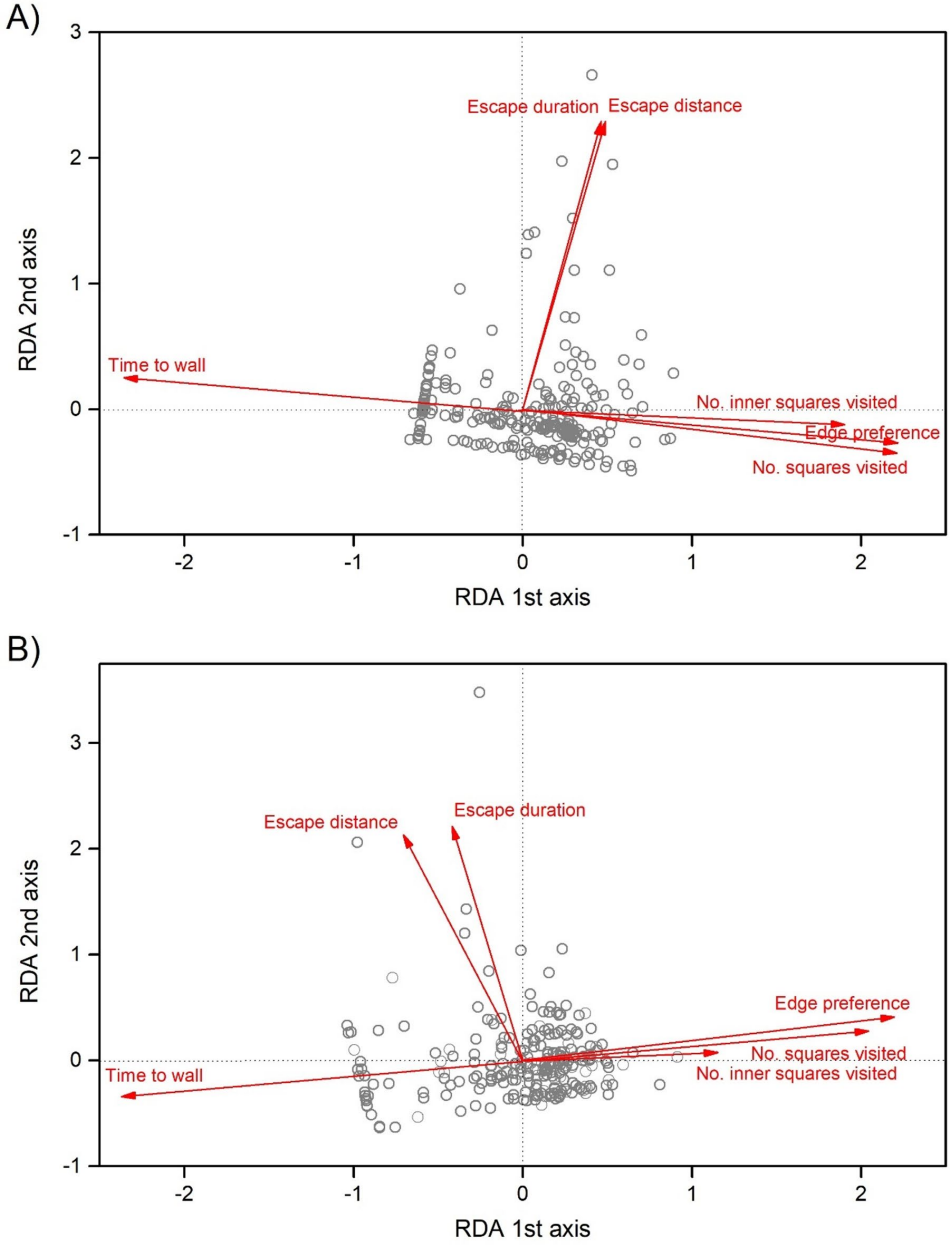
Biplot of redundancy analysis (RDA) of behavioural variation in adult *Pardosa alacris* based on six behavioural measures recorded in a novel environment test, and an escape behaviour test during the first (**A**) and second trials (**B**) 24 h apart. Grey open circles represent the 253 tested individuals, while arrows represent the six behavioural measures, with their direction and length indicating the strength and orientation of their correlations with the RDA axes (see Table 1 for details).

**Fig. 2:**
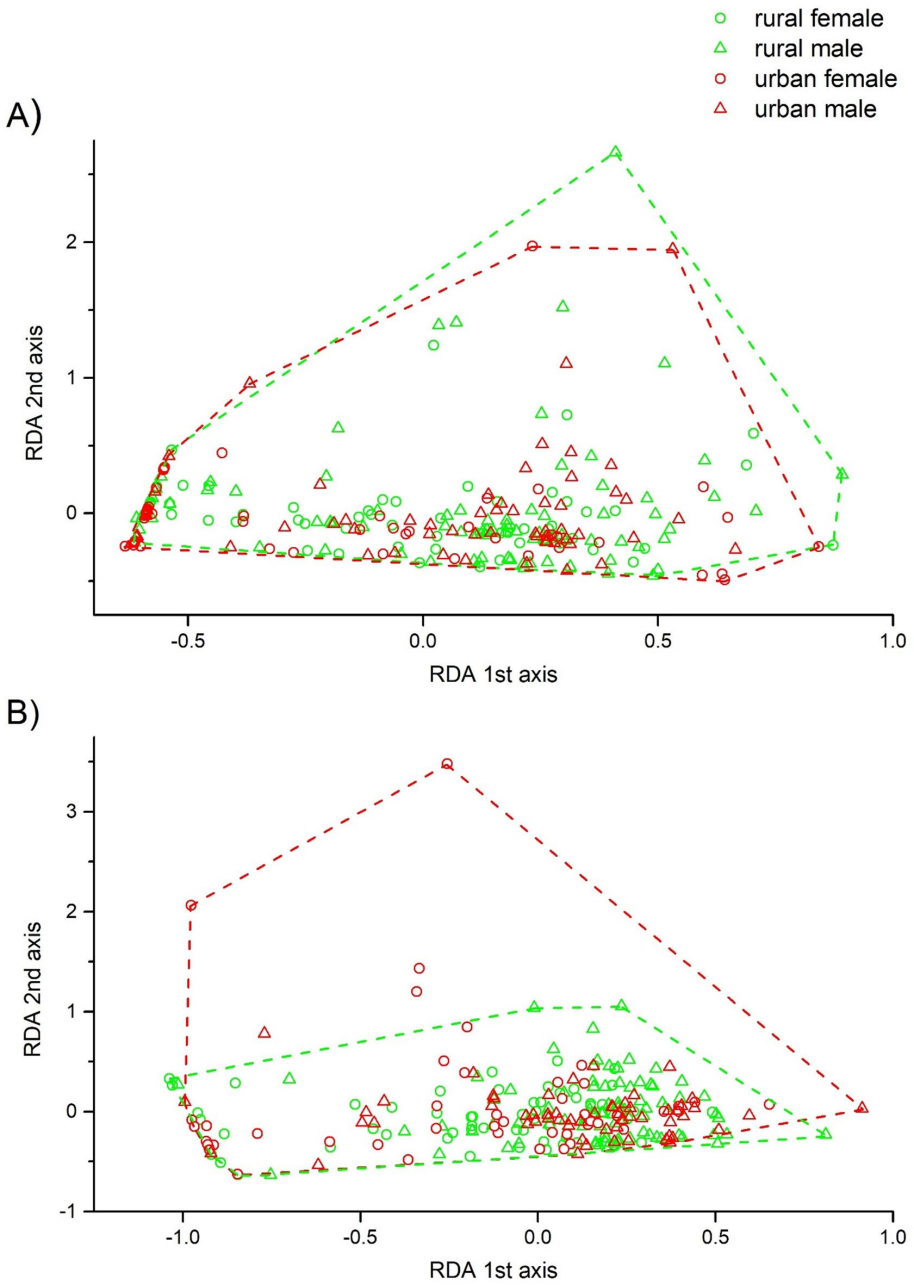
Redundancy analysis (RDA) of behavioural variation in rural vs. urban-living adult females and males of *Pardosa alacris* based on six behavioural measures recorded in a novel environment test, and escape behaviour test during the first (**A**) and second trial (**B**) 24 h apart. The dashed lines represent the convex hulls of rural and urban individuals, indicating the variation in composite behavioural scores among individuals.

The four behavioural measures recorded in a novel environment (no. squares visited, no. inner squares visited, time to wall, and edge preference; for raw data see Fig. S2) were significantly correlated with RDA axis 1, forming a composite activity-exploration-boldness behavioural score. Parameters of the escape behaviour test (escape duration and escape distance; for raw data see Fig. S3) were significantly correlated with RDA axis 2, forming the composite risk-taking behavioural score (Table 1; Fig. 1). The convex hulls of rural and urban individuals on the RDA plot were similar during the first trial (2.713 and 2.393 RDA^2^ unit for rural and urban habitats, respectively; Fig. 2). During the second trial, the convex hull was greater for urban than rural individuals (RDA^2^ unit 1.987 for rural and 4.796 for urban; Fig. 2), indicating substantial variation in the composite behavioural scores.

**Table 1:** Results of redundancy analysis (RDA) on behavioural measures on adult *Pardosa alacris* females and males collected from rural and urban habitats. Behavioural measures were recorded in two trials with 24 h apart. Loadings, eigenvalues, explained variance, and correlation coefficient (*r*^*2*^) are given for the emerging axes. Correlation between the behavioural measures and the RDA axes was tested using the *envfit* function in the *vegan* package (with 999 permutations). Correlation coefficient (*r*^*2*^) in bold denotes significant (*p* < 0.05) association with the given RDA axis. Sample size is *n* = 253 for both trials.

Behavioural measures	Loading	Correlated RDA axis	Correlation coefficient (*r*^2^)
Trial 1			
No. squares visited	0.988	RDA axis 1	**0.774**
No. inner squares visited	0.998	RDA axis 1	**0.558**
Time to wall (sec)	−0.994	RDA axis 1	**0.868**
Edge preference (sec)	0.993	RDA axis 1	**0.772**
Escape duration (sec)	0.980	RDA axis 2	**0.848**
Escape distance (no. segments)	0.979	RDA axis 2	**0.848**
Eigenvalues for the first two RDA axes	3.000 (RDA axis 1) 1.668 (RDA axis 2)		
Cumulative variance explained by the first two RDA axes (%)	77.800		
Trial 2			
No. squares visited	0.990	RDA axis 1	**0.657**
No. inner squares visited	0.997	RDA axis 1	**0.204**
Time to wall (sec)	−0.990	RDA axis 1	**0.882**
Edge preference (sec)	0.982	RDA axis 1	**0.771**
Escape duration (sec)	0.983	RDA axis 2	**0.783**
Escape distance (no. segments)	0.950	RDA axis 2	**0.779**
Eigenvalues for the first two RDA axes	2.558 (RDA axis 1) 1.518 (RDA axis 2)		
Cumulative variance explained by the first two RDA axes (%)	67.940		

Individual spiders were similarly ranked by composite behavioural scores in both trials (Kendall’s coefficient of concordance, W = 0.166, χ^2^ = 41.933, df = 1, *p* < 0.0001 for trial 1, and W = 0.118, χ^2^ = 29.917, df = 1, *p* < 0.0001 for trial 2), indicating the presence of behavioural structures. Composite activity-exploration-boldness behavioural scores were significantly repeatable when sexes were considered together or separately, and also for rural and urban spiders, indicating that spiders behaved similarly across the two trials (Table 2). However, the activity-exploration-boldness-related behaviour in urban spiders was more repeatable than in rural ones (Table 2). On the other hand, composite risk-taking behavioural scores were not significantly repeatable (Table 2).

**Table 2:** Adjusted repeatability (*R*) of the composite behavioural scores of adult *Pardosa alacris*, as well as females and males collected from rural and urban habitats. Composite behavioural scores were derived from redundancy analysis (RDA) on behavioural measures in a novel environment and an escape behaviour test. Behavioural measures recorded in a novel environment (no. squares visited, no. inner squares visited, time to wall, and edge preference) were significantly correlated with RDA axis 1, forming the composite activity-exploration-boldness behavioural score, while those evaluated in an escape behaviour test were significantly correlated with RDA axis 2, forming the composite risk-taking behavioural score (see Table 1). Values in bold denote significant (*p* < 0.05) repeatability.

Response variable	*R* [95% CI]*
All individuals	
Score on RDA axis 1	**0.219 [0.096, 0.326]**
Score on RDA axis 2	0.026 [0, 0.142]
Females	
Score on RDA axis 1	**0.228 [0.039, 0.380]**
Score on RDA axis 2	0.035 [0, 0.199]
Males	
Score on RDA axis 1	**0.195 [0.01, 0.329]**
Score on RDA axis 2	0 [0, 0.145]
Rural individuals	
Score on RDA axis 1	**0.141 [0, 0.296]**
Score on RDA axis 2	0.084 [0, 0.248]
Urban individuals	
Score on RDA axis 1	**0.286 [0.107, 0.456]**
Score on RDA axis 2	0 [0, 0.172]

*Confidence intervals (CI) were calculated using 1000 bootstraps.

**Fig. 3:**
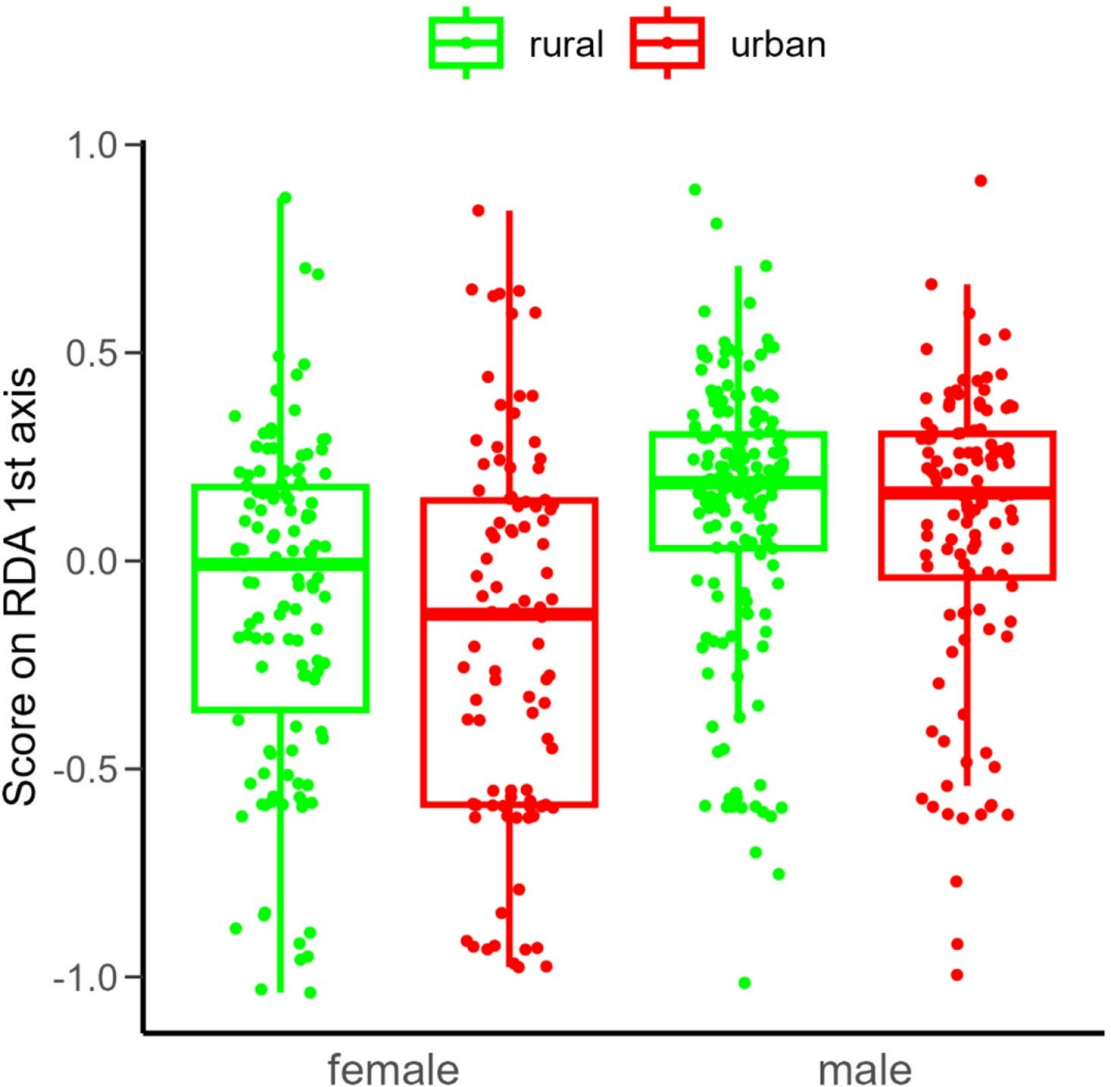
Boxplot of composite behavioural scores derived from the first redundancy analysis (RDA) axis of rural vs. urban-living adult females and males of *Pardosa alacris*. Behavioural measures recorded in a novel environment (no. squares visited, no. inner squares visited, time to wall, and edge preference) were significantly correlated with RDA axis 1, forming the composite activity-exploration-boldness behavioural score (see Table 1 for details). In boxplots the horizontal lines represent median values, the boxes denote interquartile ranges, whiskers show minimum and maximum values, while data points outside the whiskers are outliers.

Body mass had no significant effect on the composite behavioural scores, either separately or in interaction with habitat type and sex (Table S3). Females were significantly heavier than males, but there were no significant differences in body mass either at habitat level (rural vs. urban), or at habitat × sex level (Table S4 and Fig. S4). *P. alacris* males were significantly more active, exploratory and bolder than females (Table 3, and Fig. 3). Neither the habitat type (rural vs. urban), nor the habitat × sex interaction were significant factors explaining difference in composite activity-exploration-boldness behavioural score. Regarding the composite risk-taking behavioural scores, neither habitat type, sex nor their interaction were significant explanatory variables (Table 3; Fig. 3).

**Table 3:** Summary of linear mixed models on the composite behavioural scores of rural vs. urban-living adult *Pardosa alacris*. Composite behavioural scores were derived from redundancy analysis (RDA) on behavioural measures recorded for a novel environment test and an escape behaviour test. Behavioural measures recorded in a novel environment (no. squares visited, no. inner squares visited, time to wall, and edge preference) were significantly correlated with RDA axis 1, forming the composite activity-exploration-boldness behavioural score, while those evaluated in an escape behaviour test were significantly correlated with RDA axis 2, forming the composite risk-taking behavioural score (see Table 1). For the Wald χ^2^ test the degrees of freedom = 1 in all cases.

Response variable	Explanatory variable	Estimate ± SE	χ^2^	*p*
Score on RDA axis 1 $R_{m}^{2} = 0.089$ $R_{c}^{2} = 0.285$	Habitat [urban]	−0.094 ± 0.089	1.117	0.291
	Sex [male]	0.216 ± 0.046	22.388	**< 0.0001**
	Habitat [urban] × Sex [male]	0.041 ± 0.071	0.325	0.569
Score on RDA axis 2 $R_{m}^{2} = 0.005$ $R_{c}^{2} = 0.203$	Habitat [urban]	0.083 ± 0.090	0.855	0.355
	Sex [male]	0.057 ± 0.047	1.478	0.224
	Habitat [urban] × Sex [male]	−0.091 ± 0.073	1.553	0.213

Significant values (*p* < 0.05) are in bold.

## Discussion

### Composite behavioural scores

Individual behavioural measures for quantifying behavioural traits are often correlated and represent overlapping aspects of behaviour. Analysing them separately can lead to redundant tests and obscure biologically meaningful patterns^42^. For example, the time to reach the arena wall in an unfamiliar experimental arena, representing a novel environment has been used to quantify both exploration^29^ and boldness^28^, leading to conceptual ambiguity. Ordination-based composite behavioural axes integrate multiple variables into a reduced number of independent dimensions, providing a more comprehensive and interpretable representation of behavioural variation^43,44^. Consequently, these composite measures offer a robust framework for comparing behavioural traits classified according to various grouping factors (e.g. life stage, habitats, etc.). Using this approach, we identified two composite variables, the first related to activity, exploration and boldness and a second related to risk-taking. The positive correlation found between edge preference and the number of inner squares visited seems to be surprising, particularly given that wall-following activity is often interpreted as shyness^26,30^, while activity in the centre as boldness^28^. However, this is an artefact: less active individuals take longer to reach the arena wall, thereby spending more time in the central squares. Therefore, in future studies where these measures are analysed simultaneously, it is strongly recommended that the time (or its proportion) spent close to the arena wall is calculated only after individuals first reach a wall-bordering square.

## Personality versus short-term behavioural consistency

Animal personality has been widely investigated in the last three decades^4,42^ although with an emphasis on vertebrates. Arthropods have been relatively understudied despite their vast species diversity^16^. Behavioural traits associated with personality are frequently correlated and clustered, forming behavioural syndromes^42,45^. For example, bold individuals are often also more exploratory, and this behavioural syndrome is observed both in predator-exposure and foraging contexts^5^. Behavioural syndromes can potentially maintain behavioural variation among individuals in a variable environment.

Our results showed that in *P. alacris*, the composite activity-exploration-boldness scores were repeatable. Meta-analytic evidence suggests that repeatability estimates are higher when observations are taken close to each other in time^31^. However, such short intervals can overestimate behavioural stability by conflating transient state effects, such as hunger, stress, or carryover from the first trial^31^. Due to these potential complicating factors, our data do not provide a robust test of personality *sensu stricto*. Furthermore, although the composite activity-exploration-boldness behavioural scores were significantly repeatable, their effect sizes were lower than a meta-analysis-based suggestion of 0.35 ^31^ which justifies caution.

Earlier studies on spiders using longer intervals (7 days) between the repeated trials, shows that exploratory behavioural measures are repeatable over time, indicating the existence of personality *sensu stricto*^42^ in *Pardosa saltans* (Lycosidae)^46^, *Marpissa muscosa* (Salticidae)^47^ and *Larinioides sclopetarius* (Araneidae)^48^. Similarly, such time-consistent behaviour occurs in ants^49^, mantids^50^, cockroaches^51^, ground beetles^26,27^, rove beetles^24^, leaf beetles^28^ and firebugs^29^.

Composite risk-taking behavioural scores were not significantly repeatable. Such tests are routinely done by a mechanical provocation, and this is intended as a threat, to which the subject is expected to react by escaping. Some arthropods behave as expected^26,27^ but others do not^24^. During our experiments, most tested spiders escaped, but several others turned toward the forceps and attacked it. Therefore, we cannot recommend to use such “simulated attacks” to test for risk-taking behaviour or boldness, although it might possibly be suitable as a measure of aggressivity. Novel environments, such as the “open-field” test^48^ ought to be used instead, or tests more suitable for studying risk-taking behaviour and boldness (e.g. emerging from a refuge^29^).

In the behavioural ecology literature, it is widely accepted that male behaviour is more repeatable, partly because testosterone can cause males to be more predictable than females^31,52^, but also because sexually selected behavioural traits influence mate choice^31^. However, according to a recent meta-analysis^31^ other behaviours are more repeatable in females than males, challenging earlier suggestions of increased repeatability in male behaviour. We found equal repeatability between the sexes but with moderate effect size values. Similarly, there is no difference in repeatability between sexes in boldness of the raft spider, *Dolomedes fimbriatus*^53^. However, in the bridge spider, *L. sclopetarius*, activity and exploration in a novel environment are significantly repeatable only for males, while boldness is significantly repeatable for both sexes^48^. It seems that male behaviour is generally not more repeatable in spiders and substantial interspecific variation may exist. Consequently, future studies investigating spider behaviour should explicitly consider and test for the effects of sex.

### Differences in behaviour between males and females

Males of *P. alacris* were more active, exploratory and bolder than females. Such sex-dependent differences can arise from distinct life-history strategies and differential selective pressures. Males generally actively search for a mating partner^54^, underlining different reproductive strategies and reproductive investments as a consequence of anisogamy. Females focus heavily on egg production, while males try to maximise mating opportunities, with consequent dissimilarities in activity, exploration and boldness. Sex-specific selection pressures driven by predation risk and sexual cannibalism^55^ could also be a reason for sex-dependent differences in behaviour^56^.

Males of other wolf spiders, including *P. saltans*^46^ and *Schizocosa ocreata*^57^ are significantly more active and exploratory than females. Similarly, immature males of a protandrous jumping spider, *Carrhotus xanthogramma* are more active and more risk tolerant than females^58^. However, there are no significant sex-dependent differences in behavioural measures in a novel environment either in jumping spider, *M. muscosa*^47^ or in the bridge spider, *L. sclopetarius*^48,59^. Contrary to our results, in raft spider, *D. fimbriatus*, females are bolder and show higher overall behavioural responsiveness than males^53^. *D. fimbriatus* females are selected for increased fecundity, and exhibit consistently high feeding rates throughout their lifespan, enabling egg production. However, yolk is incorporated into the eggs only upon maturation, requiring females to forage intensively throughout the reproductive period^53^. In contrast, male *D. fimbriatus* benefit from increased foraging and growth during juvenile and subadult stages, whereas adult males substantially decrease foraging and primarily invest energy in competition with rival males and reproductive behaviour^53^. These patterns highlight that spider behaviour is shaped by species-specific selective pressures. Consequently, studies across a broad range of species are required to identify and disentangle the full set of factors influencing behavioural variation and personality in spiders.

### Urbanisation and behavioural traits

Anthropogenic disturbances (e.g. traffic, impervious surfaces, various pollutants) and environmental changes that accompany urbanisation create new challenges and often a novel environment for animals. Some behavioural traits may be advantageous for coping with these. Most behavioural studies were carried out on vertebrates (frequently on birds^60–62^, and mammals^63–65^), and show that urban-living individuals are bolder^60,62–64^ and more exploratory^61,63^ than their rural conspecifics. Similarly, several urban arthropods, including grasshoppers^66^, ants^67,68^, beetles^14^ are bolder and/or exploratory and/or more active than their conspecifics from natural habitats.

This is, however, not a general rule. Urban *P. alacris* adults did not differ either in activity-exploration-boldness or the composite risk-taking behavioural scores from rural ones. Similar results were reported for the western black widow spider, *Latrodectus hesperus*^69^. In contrast, another study on three *Latrodectus* spp. found that urban individuals of one of them, *L. sclopetarius* exhibits higher activity in a novel environment than the other two from suburban habitats^9^. Since measures of behavioural traits can vary significantly even among species belonging to the same taxonomic group^70^, this result should be treated with caution, because the comparisons are not within the same species^9^. There are several studies that do not show significant behavioural differences between rural and urban arthropods^24,27,71^. Our results, which did not show significant differences in behaviour between rural and urban individuals, along with the inconclusive findings of previous studies, suggest that urbanisation does not necessarily trigger increased exploratory and risk-taking behaviour in arthropods.

Given that urbanisation is an evolutionarily very recent phenomenon, it is possible that urban-adapted genotypes could not have been selected. The short generation time of *P. alacris* may allow selection to still occur, and urban populations may have undergone genetic differentiation if sufficiently isolated from rural ones^72^. However, spiders use silk to increase their dispersal capacity, so continued immigration and emigration^73^ between habitats is likely, diluting any effects of the selection pressure posed by local conditions.

The relatively low marginal and conditional *R*^2^ values in our models indicated that broad habitat categories and sex explained only a limited proportion of the observed behavioural variation. This suggests that spider behaviour is largely shaped by fine-scale environmental heterogeneity and individual-level differences rather than by differences in habitat conditions alone^74^. Such low explanatory power of fixed effects may be common in behavioural ecology and reflects the inherently context-dependent and state-dependent nature of behavioural traits^75^. Future studies would therefore benefit from incorporating relevant, continuous environmental variables (e.g. microclimatic conditions, micro-habitat structure, or resource availability), as well as additional individual-based variables beyond body mass, such as reproductive stage (pre- or post-mating stage), or nutritional status.

Although mean behavioural measures did not differ significantly between urban and rural individuals, substantially different inter-individual variation may exist between individuals living in the two habitat types^9,48^, contributing to adaptive responses to environmental challenges^76^. In the present study, using the area of the convex hulls in the RDA plot, we also confirmed a substantial between-individual variation in the studied behavioural variables. We showed higher between-individual variation in the composite behavioural scores, especially in the risk-taking behavioural one, for urban than rural spiders during the second trial. This difference was detected only during the second trial which highlights that the stress associated with trapping and laboratory transport can have a substantial effect on behaviour. Providing experimental animals with a resting or acclimation period of at least 24 h following laboratory transfer seems advisable.

Besides higher among individual variation in behaviour of urban than rural spiders, activity-exploration-boldness-related behaviour of urban individuals were also more repeatable than that of rural ones. This suggests that urban spiders exhibit more consistent and diversified behavioural strategies. Consistent individual differences are expected to emerge when environmental conditions are relatively predictable and more limiting, and under such conditions, consistent behaviour may be favoured by selection^4,77^. In contrast, rural habitats are typically more heterogeneous and variable in space and time, which may favour behavioural flexibility over consistency. When the costs and benefits of behaviours fluctuate across contexts, selection may favour individuals that adjust their behaviour according to current environmental or physiological conditions rather than expressing stable behavioural phenotypes^4^. Furthermore, differences in repeatability may reflect habitat-specific risk-reward trade-offs: in urban habitats, bold or exploratory behaviour may have lower relative costs, allowing such strategies to persist consistently, whereas in rural habitats the same behaviours may cause higher or more variable risks, promoting plasticity instead of stable behavioural syndromes^77,78^.

## Conclusions

We can conclude that short-term behavioural consistency in activity, exploration and boldness exist in *P. alacris*. Urbanisation did not change this aspect of behaviour in either males or females, presumably because their good dispersal ability allowed them to settle quickly in suitable microhabitats, even in dramatically modified urban environments. Thus, increased exploration or risk taking would not offer further fitness benefits. However, males from both habitats were more active, exploratory, and bolder than females, possibly because their distinct life-history strategies and sex-specific selective pressures. Furthermore, it would be useful to evaluate seasonal differences (e.g. behaviour patterns during the mating and post-mating periods) since the success of mating can also be influenced by the studied behavioural traits, with consequences for individual fitness.

## Supplementary Information

Below is the link to the electronic supplementary material.

Supplementary Material 1

## Data Availability

Data used for analyses are available in the Mendeley repository (doi: 10.17632/j6x8hyxgj9.2; https://data.mendeley.com/datasets/j6x8hyxgj9/2).
