# Supplementary material for: Sex-specific but not urbanisation-related behavioural differences in a wolf spider, Pardosa alacris
Source: Sci Rep. 2026 Mar 5;16:12253. doi: 10.1038/s41598-026-41239-2 (PMC13079826; doi:10.1038/s41598-026-41239-2)
Supplement: Supplementary file 1 — Supplementary Material 1 [file 41598_2026_41239_MOESM1_ESM.docx]

**SUPPLEMENTARY INFORMATION**

Magura T, Horváth R, Mizser S, Tóth M, Kozma FS, Lövei GL.

Sex-specific but not urbanisation-related behavioural differences in a wolf spider, *Pardosa alacris*.

*Scientific Reports*

**Table S1**. The number of sampled adult *Pardosa alacris* individuals in the rural and urban sites between late March and end of June, 2020.

| **Site** | **All individuals** | **Females** | **Males** |
| --- | --- | --- | --- |
| Rural site 1 | 34 | 15 | 19 |
| Rural site 2 | 39 | 16 | 23 |
| Rural site 3 | 39 | 15 | 24 |
| Rural site 4 | 36 | 17 | 19 |
| All rural sites | 148 | 63 | 85 |
| Urban site 1 | 38 | 19 | 19 |
| Urban site 2 | 22 | 6 | 16 |
| Urban site 3 | 27 | 12 | 15 |
| Urban site 4 | 18 | 10 | 8 |
| All urban sites | 105 | 47 | 58 |
| All sites | 253 | 110 | 143 |

**Table S2.** Spearman rank-correlations between the tested behavioural measures (average of the two trials) of *Pardosa alacris* individuals collected in rural and urban habitats. Values in bold denote significant (*p* < 0.05) correlations.

|  | No. squares visited | No. inner squares visited | Time to wall | Edge preference | Escape duration | Escape distance |
| --- | --- | --- | --- | --- | --- | --- |
| No. squares visited | 1.0000 | **0.6412** | **-0.6492** | **0.5526** | 0.0279 | -0.0132 |
| No. inner squares visited | **0.6412** | 1.0000 | **-0.4654** | **0.3355** | 0.0272 | -0.0246 |
| Time to wall | **-0.6492** | **-0.4654** | 1.0000 | **-0.9275** | -0.0383 | 0.0175 |
| Edge preference | **0.5526** | **0.3355** | **-0.9275** | 1.0000 | 0.0387 | -0.0191 |
| Escape duration | 0.0279 | 0.0272 | -0.0383 | 0.0387 | 1.0000 | **0.6695** |
| Escape distance | -0.0132 | -0.0246 | 0.0175 | -0.0191 | **0.6695** | 1.0000 |

**Table S3.** Summary of linear mixed models on the composite behavioural scores of adult *Pardosa alacris* females and males sampled from rural and urban habitats. Behavioural scores were derived from a redundancy analysis (RDA) on behavioural measures recorded for a novel environment and escape behaviour. Behavioural measures recorded in a novel environment (no. squares visited, no. inner squares visited, time to wall, and edge preference) were significantly correlated with RDA axis 1, forming the composite activity-exploration-boldness behavioural score, while those evaluated in the escape behaviour test were significantly correlated with RDA axis 2, forming the composite risk-taking behavioural score (see Table 1). For the Wald χ^2^ tests, the degrees of freedom = 1 in all cases.

| **Response variable** | **Explanatory variable** | **Estimate ± SE** | **χ^2^** | ***p*** |
| --- | --- | --- | --- | --- |
| ***Score on RDA axis 1***  $R_{m}^{2}=0.111$  $R_{c}^{2}=0.302$ |  |  |  |  |
|  | Habitat [urban] | -0.187 ± 0.192 | 0.944 | 0.331 |
|  | Sex [male] | 0.205 ± 0.164 | 1.579 | 0.209 |
|  | Mass | 8.380 ± 5.621 | 2.222 | 0.136 |
|  | Habitat [urban] × Sex [male] | 0.117 ± 0.246 | 0.227 | 0.634 |
|  | Habitat [urban] × Mass | 5.418 ± 8.600 | 0.397 | 0.529 |
|  | Sex [male] × Mass | 13.983 ± 13.918 | 1.009 | 0.315 |
|  | Habitat [urban] × Sex [male] × Mass | -2.663 ± 21.920 | 0.015 | 0.903 |
| ***Score on RDA axis 2***  $R_{m}^{2}=0.009$  $R_{c}^{2}=0.206$ |  |  |  |  |
|  | Habitat [urban] | -0.075 ± 0.198 | 0.143 | 0.705 |
|  | Sex [male] | -0.133 ± 0.169 | 0.621 | 0.431 |
|  | Mass | -3.187 ± 5.811 | 0.301 | 0.583 |
|  | Habitat [urban] × Sex [male] | 0.162 ± 0.253 | 0.408 | 0.523 |
|  | Habitat [urban] × Mass | 7.944 ± 8.860 | 0.804 | 0.370 |
|  | Sex [male] × Mass | 18.161 ± 14.376 | 1.596 | 0.207 |
|  | Habitat [urban] × Sex [male] × Mass | -19.184 ± 22.517 | 0.726 | 0.394 |

**Table S4.** Summary of linear mixed models on the body mass of adult *Pardosa alacris* individuals sampled from rural and urban habitats (*p* values in bold denote significant (p < 0.05 effect). For the Wald χ^2^ tests, the degrees of freedom = 1 in all cases.

| **Response variable** | **Explanatory variable** | **Estimate ± SE** | **χ^2^** | ***p*** |
| --- | --- | --- | --- | --- |
| ***Body mass***  $R_{m}^{2}=0.642$  $R_{c}^{2}=0.651$ |  |  |  |  |
|  | Habitat [urban] | -0.001 ± 0.001 | 3.441 | 0.064 |
|  | Sex [male] | -0.013 ± 0.001 | 582.406 | **< 0.0001** |
|  | Habitat [urban] × Sex [male] | 0.001 ± 0.001 | 2.158 | 0.142 |

| A)  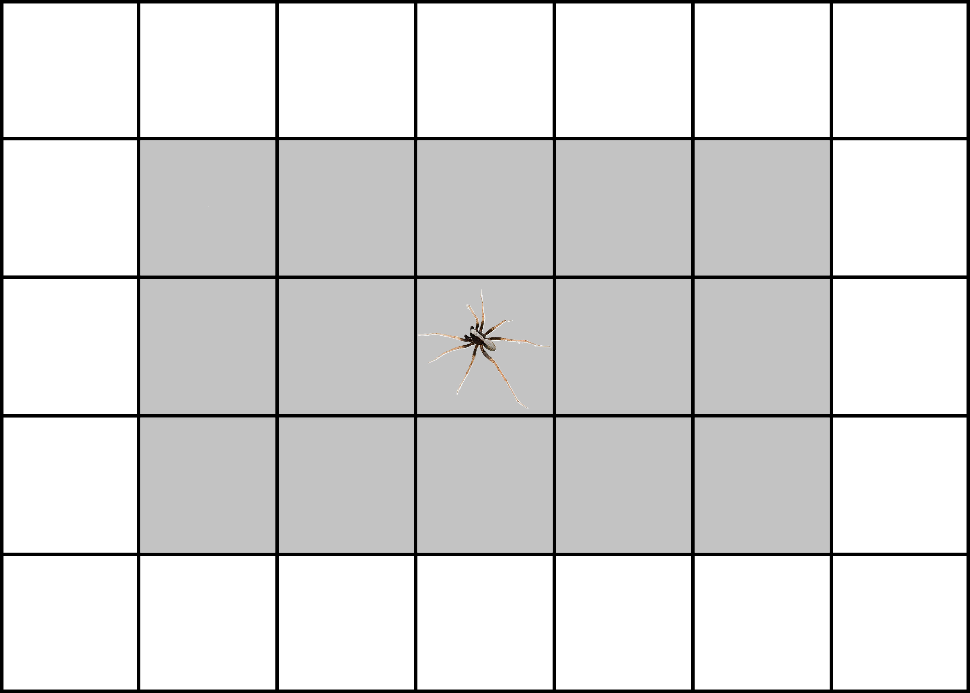  10 cm |
| --- |
| B)  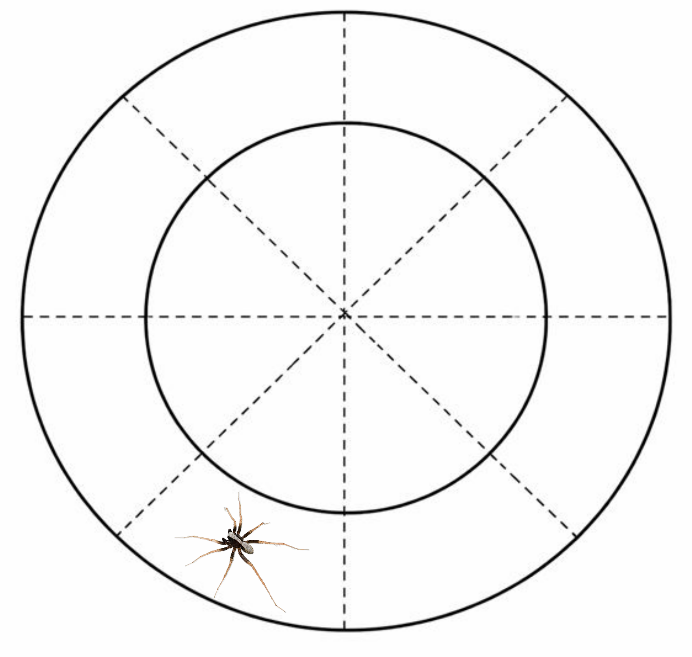  10 cm |

**Figure S1**. Schematic image of (A) the novel environment arena, with inner squares marked by grey, and (B) the ring-shaped arena for testing the escape behaviour.


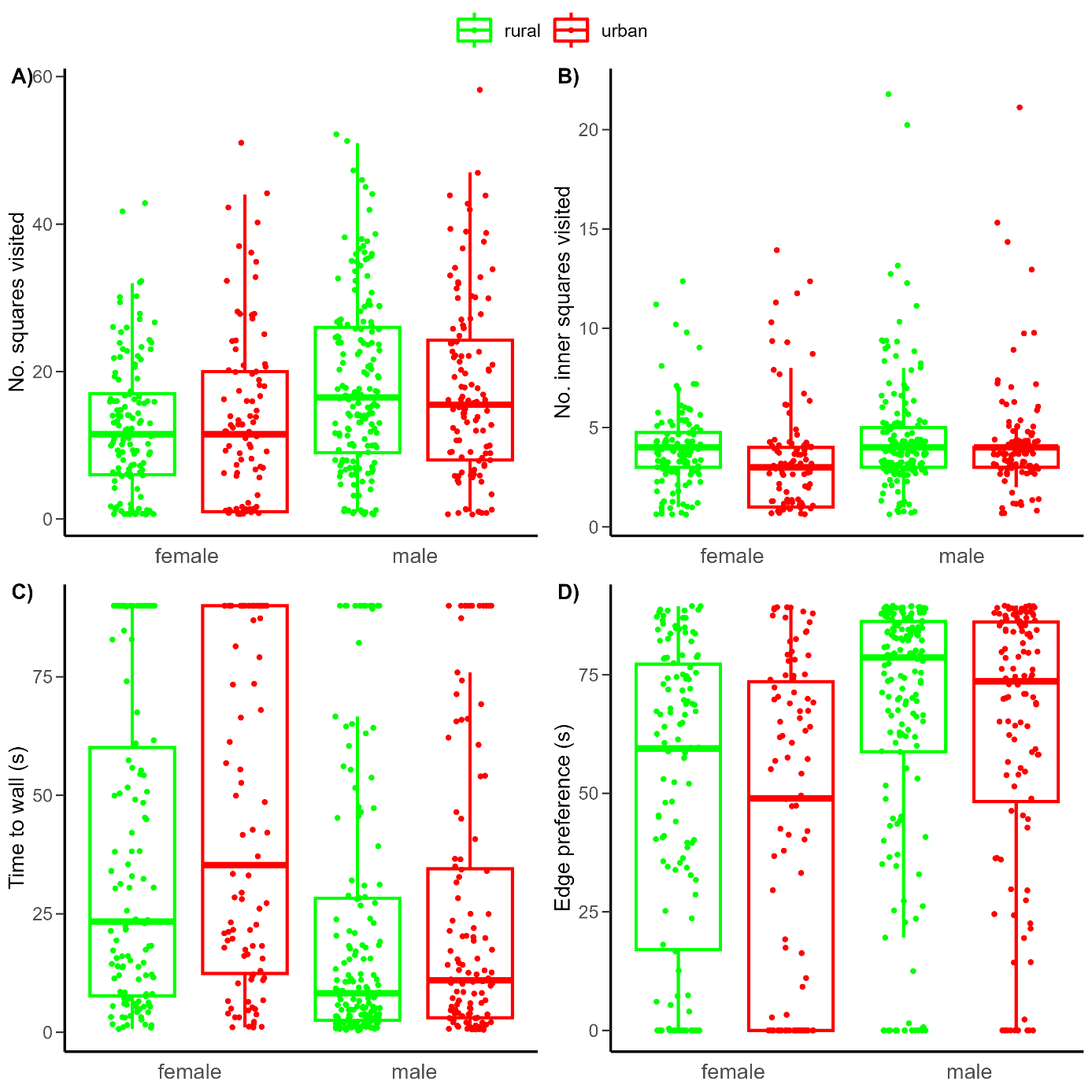


**Figure S2.** Boxplot of behavioural measures recorded from novel environment tests of *Pardosa alacris* female and male individuals sampled from rural and urban habitats: (A) the number of squares covered by individuals (no. squares visited), (B) the number of squares not adjacent to the wall of the arena entered by individuals (no. inner squares visited), (C) the time (s) when the individuals reached the wall of the arena (time to wall), and (D) the time (s) individuals spent in the squares adjacent to the wall of the arena (edge preference). In boxplots the horizontal lines represent median values, the boxes denote interquartile ranges, whiskers show minimum and maximum values, while data points outside the whiskers are outliers.


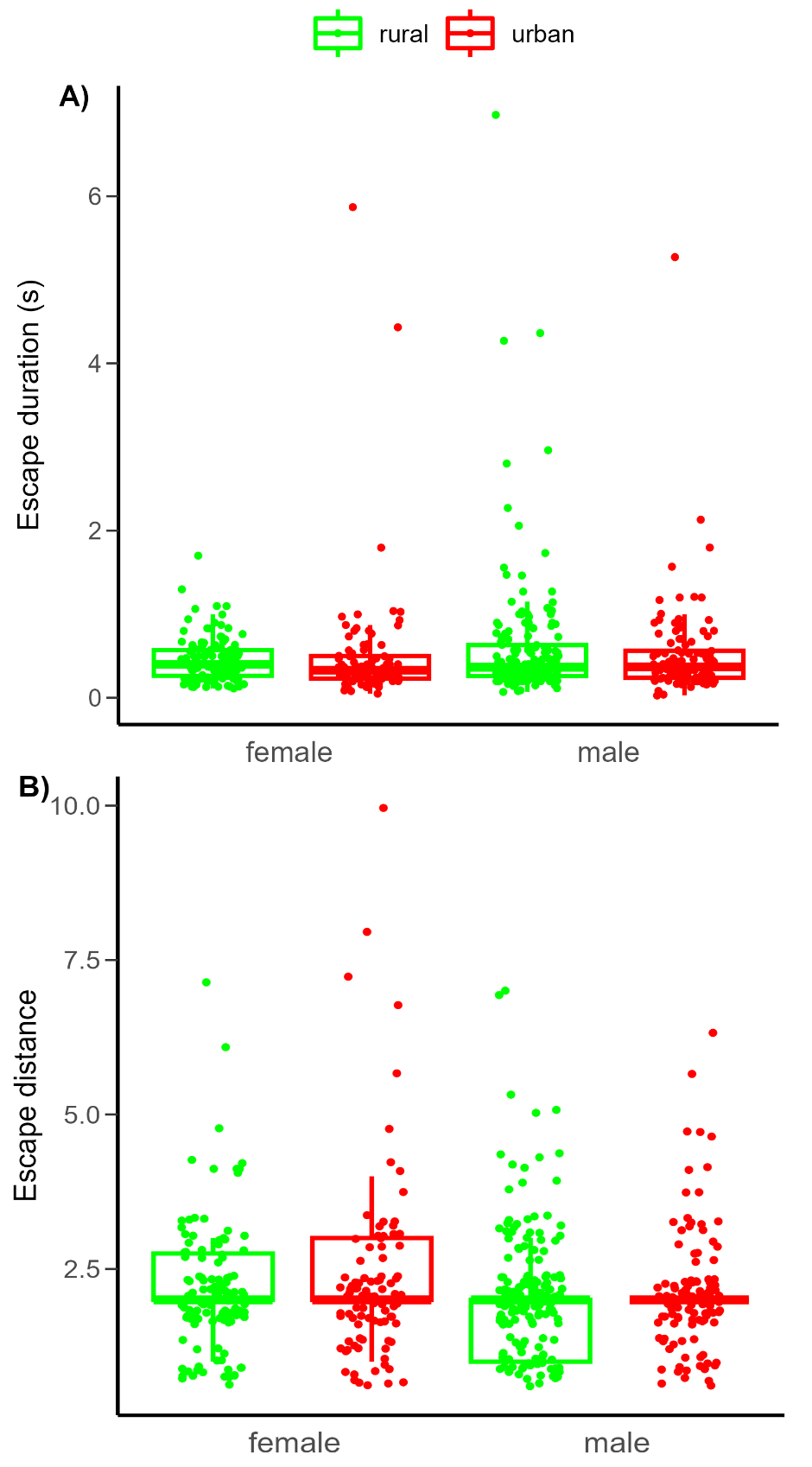


**Figure S3.** Boxplot of behavioural measures recorded from escape behaviour tests of *Pardosa alacris* female and male individuals sampled from rural and urban habitats: (A) the time (s) spent running after a mechanical stimulus (escape duration), and (B) the number of segments crossed during the fleeing (escape distance). In boxplots the horizontal lines represent median values, the boxes denote interquartile ranges, whiskers show minimum and maximum values, while data points outside the whiskers are outliers.


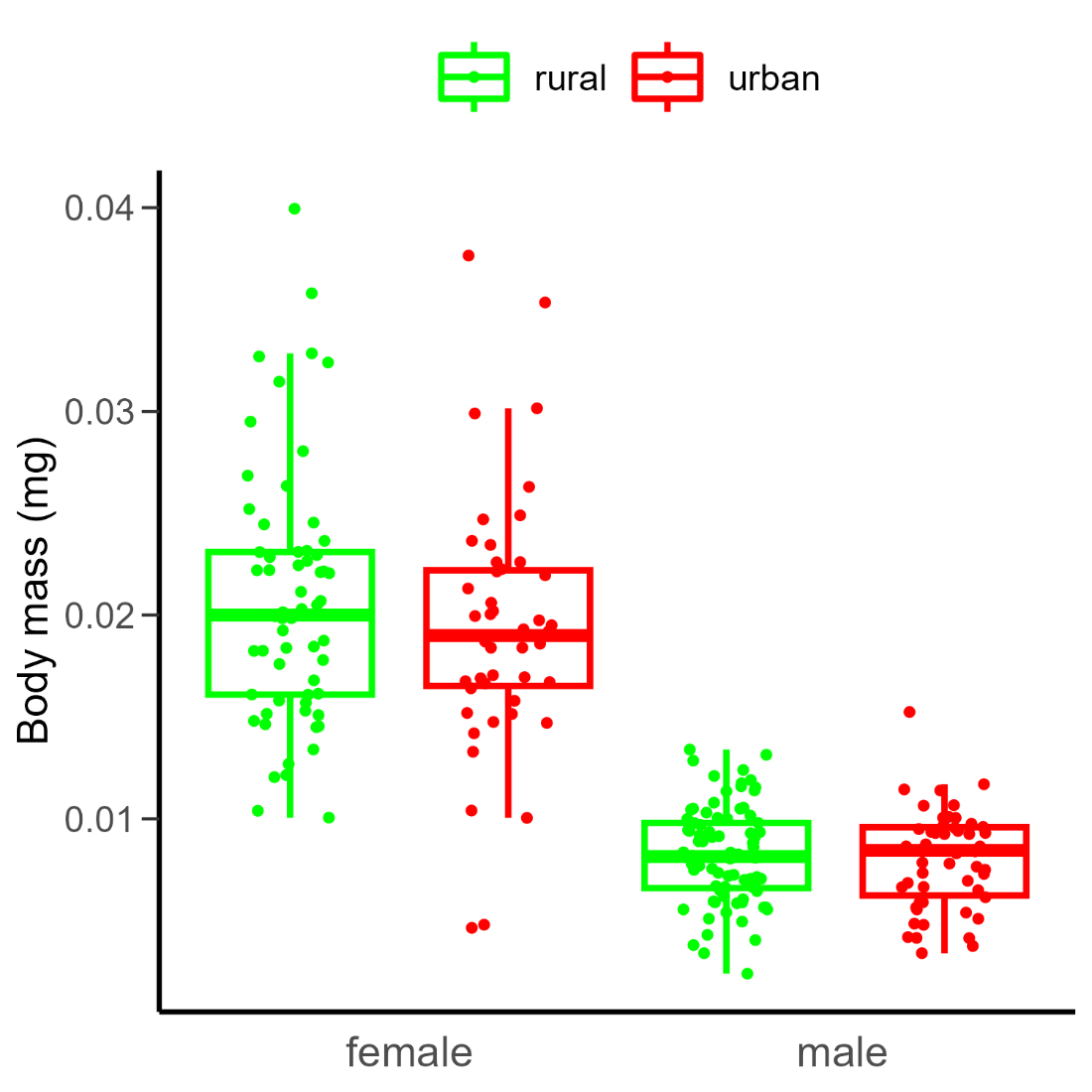


**Figure S4.** Boxplot of body mass (mg) values of *Pardosa alacris* female and male individuals collected from rural and urban habitats. In boxplots the horizontal lines represent median values, the boxes denote interquartile ranges, whiskers show minimum and maximum values, while data points outside the whiskers are outliers.
